# CO_2_ Supercritical Fluid Extraction of Oleoresins from Sea Buckthorn Pomace: Evidence of Advanced Bioactive Profile and Selected Functionality

**DOI:** 10.3390/antiox10111681

**Published:** 2021-10-25

**Authors:** Liliana Mihalcea, Mihaela Turturică, Elena Iulia Cucolea, George-Mădălin Dănilă, Loredana Dumitrașcu, Gigi Coman, Oana Emilia Constantin, Leontina Grigore-Gurgu, Nicoleta Stănciuc

**Affiliations:** 1 Faculty of Food Science and Engineering, Dunărea de Jos University of Galati, Domnească Street 111, 800201 Galati, Romania; Liliana.Gitin@ugal.ro (L.M.); Mihaela.Turturica@ugal.ro (M.T.); Loredana.Dumitrascu@ugal.ro (L.D.); Gigi.Coman@ugal.ro (G.C.); Emilia.Constantin@ugal.ro (O.E.C.); Leontina.Gurgu@ugal.ro (L.G.-G.); 2 Cromatec Plus SRL, Research Center for Instrumental Analysis SCIENT, Petre Ispirescu Street 1, 077176 Tâncăbești, Romania; iulia.cucolea@scient.ro (E.I.C.); madalin.danila@scient.ro (G.-M.D.)

**Keywords:** sea buckthorn pomace, oleoresins, CO_2_ supercritical fluid extraction, antioxidant activity, antimicrobial activity

## Abstract

The processing of sea buckthorn generates a significant amount of pomace, seeds and skin considered valuable sources of health-promoting macromolecules, such as carotenoids, pectin, flavonoids, phytosterols, polyunsaturated fatty acids and tocopherols. In this study, the bioactives from sea buckthorn pomace (SBP) were extracted using supercritical carbon dioxide (SFE-CO_2_), at different temperatures and pressures, allowing for obtaining four fractions according to separators (S40 and S45). The highest carotenoid content of 396.12 ± 1.02 mg/g D.W. was found in the S40 fraction, at extraction parameters of 35 °C/45 MPa, yielding an antioxidant activity of 32.10 ± 0.17 mMol TEAC/g D.W. The representative carotenoids in the extract were zeaxanthin, β-carotene and lycopene, whereas all enriched SFE-CO_2_ extracts contained α-, β- and δ-tocopherol, with α-tocopherol representing around 82% of all fractions. β-sitosterol was the major phytosterol in the fractions derived from S45. All fractions contained significant fatty acids, with a predominance of linoleic acid. Remarkably, the enriched extracts showed a significant palmitoleic acid content, ranging from 53 to 65 µg/g. S40 extracts showed a good antibacterial activity against *Staphylococcus aureus* and *Aeromonas hydrophila* ATCC 7966, whereas S45 extracts showed a growth inhibition rate of 100% against *Aspergillus niger* after three days of growth. Our results are valuable, and they allow identifying the different profiles of extracts with many different applications in food, pharmaceutics, nutraceuticals and cosmeceuticals.

## 1. Introduction

Significant health consequences associated with an increase in obesity and chronic ailments, such as cardiovascular diseases and cancer, are determined by major shifts in humans’ dietary patterns accompanying rapid nutritional transitions [1]. Therefore, an important aspect in circumventing the detrimental effects of nutritional transitions is developing and promoting functional foods, especially food enriched with biologically active compounds, with high nutraceutical and functional characteristics, thus positively impacting human health and disease prevention [2].

Sea buckthorn (*Hippophaë rhamnoides* L.) belongs to the Elaeagnaceae family, with small oval-shaped berries, mostly orange-yellow or orange-red [3]. The berries are rich sources of nutrients and bioactives, including vitamins, carotenoids (β-carotene, zeaxanthin, lutein, lycopene, etc.), polyphenols, flavonoids, organic acids, pectin, carbohydrates, polyunsaturated fatty acids and essential amino acids [4]. These compounds are intensively studied for their related radioprotection, antibacterial, anti-stress, immunity regulation, free radical scavenging, cardiovascular disease and cancer prevention and tissue regeneration abilities [5,6]. For example, Tanaka, Shnimizu and Moriwaki [7] suggested that carotenoids such as β-carotene, α-carotene, lycopene, lutein, zeaxanthin, β-cryptoxanthin, fucoxanthin, canthaxanthin and astaxanthin have anti-carcinogenic activity in several tissues. However, berries are very acidic and perishable, being processed in different products such as jams, jellies, sweets, juice, oil, herbal teas, supplements and cosmetics, or used in traditional medicines [8]. Processing of sea buckthorn leads to a significant amount of pomace, discarded as waste, with a high pollution potential. The pomace, consisting of skin and seeds, contains a significant amount of valuable bioactives, including flavonoids, carotenoids, phenolic compounds, polyunsaturated fatty acids and vitamins, which have antibacterial and antioxidant effects and may be extracted and used in different applications [9].

In order to develop strategies for the efficient recovery of these bioactives, optimization of the extraction parameters is needed. Supercritical carbon dioxide (SFE-CO_2_) was successfully used for the extraction of bioactive compounds from blackcurrant pomace [10], raspberries [11] and black chokeberries [12], as well as from sea buckthorn seeds [13], allowing for obtaining higher-added value fractions, rich in bioactive compounds. The use of SFE-CO_2_ for bioactive extraction offers certain advantages, being considered a green extraction technology broadly applied to recover valuable bioactives from different materials, both at the laboratory and industrial levels.

Therefore, this study aimed to test two sets of selected parameters for the SFE-CO_2_ extraction of oleoresins to allow a valuable fractionation of sea buckthorn pomace (SBP) into enriched extracts. Hence, different parameters were tested, in terms of extraction temperature and pressure, whereas the extracts were assessed for global phytochemical composition (total carotenoids, β-carotene, lycopene and polyphenols) and antioxidant capacity. Advanced profiling of the selected enriched oleoresin extracts was performed based on the global phytochemical profile in terms of fatty acid, phytosterol and tocopherol contents. The functionality of the selected enriched oleoresin extracts was tested for antimicrobial activity. The obtained results are expected to provide significant evidence for the valorization of SBP for further food, nutraceutical, pharmaceutical and cosmeceutical applications.

## 2. Materials and Methods

### 2.1. Materials

SBP were obtained from a local juice producer (S.C. Bio-Hipporham SRL, Urzica Village, Olt County, Romania), dried at 35 °C in a fluidized bed dryer FT-31 model (Armfield, Ringwood, UK) and ground by a hand mill with 180 W electric power (Bosch, Gerlingen, Germany). All reagents and solvents were of analytical and HPLC grade.

### 2.2. SFE-CO_2_ Extraction

SFE-CO_2_ extractions were performed with the pilot plant equipped with an extractor vessel with a total internal volume of 2L, and its maximum operating pressure and temperature were 55 MPa and 120 °C, respectively. The equipment is provided with two separators (S40 and S45), each with a volume of 1.5 L (Natex Prozesstechnologie GesmbH, Ternitz, Austria). For each extraction, 0.400 kg of the SBP (91.34 ± 0.35% dry weight) was loaded into the extraction vessel. The air in the system was removed by flushing the system with CO_2_, and the extractor was pressurized with CO_2_ (99,999% purity supplied by Messer S.A., Bucharest, Romania) using a high-pressure CO_2_ pump. According to our preliminary kinetic studies, two combinations of temperature/pressure were tested for extraction as follows: 35 °C/45 MPa (E1) and 37.5 °C/36.5 MPa (E2). The extraction time was set at 105 min. The CO_2_ flow was monitored with a Coriolis mass flow meter and generated by the data sheets from ABB software (ABB-Mannheim, Mannheim, Germany). The average CO_2_ mass flow for the extraction batches was 21.23 ± 0.61 kg/h. In order to produce fractions with different compositions, the pressure in the first separator (S40) was maintained constant at 15 MPa, while in the second separator (S45), decompression up to a recirculation pressure of 5 MPa was set [14]. Each extract was coded according to the separators, S40 and S45, and kept in dark bottles under refrigeration conditions (2–4 °C) until use. The extraction yield was calculated as the ratio between the weight of the extracted oleoresin and the weight of matrices loaded in the extractor vessel [15].

### 2.3. Global Phytochemical Profiling

The extracts were characterized in terms of total carotenoid, β-carotene, lycopene and total polyphenolic contents using spectrophotometric methods. In general, about 5 mg of concentrated extracts was dissolved in 10 mL of a mixture of *n*-hexane/acetone (ratio of 3:1, *v*/*v*). For the carotenoid content, different wavelengths were selected: 450 nm for total carotenoid content, 470 nm for β-carotene and 503 nm for lycopene. The carotenoid amount was expressed as mg/g dry weight (DW) according to the following Equation (1):

(1) $$\mathrm{Carotenoids}\;\left(\frac{\mathrm{mg}}{g}\right)=\frac{{A\times M}_{w}{\times D}_{f}}{M_{a}\times L}$$

where A is absorbance at 470 nm, 450 nm and 503 nm; M_w_ is the molecular weight for lycopene and β-carotene (536.873 and 536.87 g/mol, respectively); D_f_ is the sample dilution rate; M_a_ is the molar absorptivity in n-hexane (3450 L/mol·cm for lycopene, 2590 L/mol·cm for total carotenoids and 2500 L/mol·cm for β-carotene); and L is the cell diameter of the spectrophotometer (1 cm).

### 2.4. Antioxidant Activity

The radical scavenging ability of the extracts was evaluated using TEAC (Trolox equivalent antioxidant capacity) by using the 2,2-azinobis-3-ethyl-benzothialzoline-6-sulfonic acid (ABTS+) radical cation decoloring reaction. Briefly, ABTS+ solution was prepared by mixing an ABTS+ aqueous solution at a concentration of 7.0 mM with potassium persulfate (2.45 mM final concentration), followed by a reaction for 16 h in the dark at room temperature. A volume of 0.15 mL of extracts, as previously described, was added to a volume of 2.85 mL of the ABTS+ solution and allowed to react for 2 h in the dark. The absorbance of the mixture was measured at 734 nm. The ABTS+ antioxidant activity of the extracts was expressed as mM TEAC/g D.W. based on the calibration curve.

### 2.5. Chromatographic Analysis of Individual Carotenoids

Chromatographic analysis, in order to characterize the individual carotenoids, was performed according to the method described by Gheonea et al. [16]. For the identification and the quantification of carotenoids from SBP oleoresins, a Thermo Finnigan Surveyor HPLC system (Finnigan Surveyor, Thermo Scientific, Waltham, MA, USA) was used, equipped with an Acclaim C30 (150 × 3 mm, 3 μm). The operating parameters were: flow rate 0.6 ml/min, 10 μL injection volume and a binary gradient consisting of 90% acetonitrile (90:10) in ultrapure water (*v*/*v*) (solvent A) and 100% ethyl acetate (*v*/*v*) (solvent B).

### 2.6. Fatty Acids

Fatty acids present in the extracts were converted into their respective methyl esters (FAMEs) using the methanolic boron trifluoride (BF3)-catalyzed esterification procedure. Briefly, 100 ± 5 mg of extract was weighed in a 50 mL round-bottom flask. Then, 4 mL of 0.5 M methanolic NaOH was added, and the mixture was refluxed for 30 min. After 30 min, 5 mL of 15% (*v*/*v*) methanolic BF3 was added to the flask and refluxed for another 3–5 min. After the samples were cooled, the organic fraction containing FAMEs was extracted 3 times with 3 mL of n-hexane and diluted to 20 mL with n-hexane as the final volume. An amount of 1 µL of FAME extract was injected in the gas chromatographic system coupled with a mass spectrometer (Perkin Elmer Clarus 680/SQ 8T, Perkin Elmer, Waltham, MA, USA) equipped with an Elite-WAX capillary column (30 m × 0.25 mm i.d., 0.25 µm film thickness (Perkin Elmer, Waltham, MA, USA)), using helium as the carrier gas (flow rate of 1.5 mL/min). The temperature program was as follows: initial temperature 100 °C, increase by 20 °C/min to 200 °C, hold time 8 min, then increase by 5 °C/min to final temperature of 250 °C and hold for 6 min. The injection volume was 1 µL in split mode (split ratio 20:1), and the injector temperature was set to 220 °C. The MS operating conditions were: source temperature 200 °C, transfer line temperature 220 °C, electron impact ionization EI+ at 70 eV and a solvent delay of 6 min. The quantification of FAMEs present in the extracts was performed in selected ion recording mode (SIR), using a 5-point calibration curve prepared from a 37-component FAME Mix (Supelco, Sigma-Aldrich, Darmstadt, Germany). The experiments were performed in duplicates.

### 2.7. Phytosterols

The phytosterol profile of SBP extracts was determined via GC-MS using the extracts dissolved in n-hexane as mentioned above. Briefly, 2 mL of extract was evaporated to dryness under a nitrogen-free oxygen stream. The residue was derivatized with 100 µL of MSTFA (Mackerey-Nagel, Dueren, Germany) at 60 °C for 45 min, and 1 µL was injected in the GC-MS system equipped with an Elite-5ms capillary column (30 m × 0.25 mm i.d., 0.25 µm film thickness of 5% phenyl-95% methylpolysiloxane, Perkin Elmer, Waltham, MA, USA), using helium as the carrier gas (flow rate 1.0 mL/min). The temperature program was: initial temperature 50 °C, hold time 1 min, increase by 20 °C/min to 230 °C, hold time 3 min, then increase by 10 °C/min to final temperature of 310 °C and hold for 3 min. The injection was performed in split mode (split ratio 10:1), and the injector temperature was set to 230 °C. The MS operating conditions were: source temperature 230 °C, transfer line temperature 240 °C, electron impact ionization EI+ at 70 eV and a solvent delay of 1.5 min. The identification of phytosterols was achieved in the full scan mode with the scanned mass range 100–600.

### 2.8. Tocopherols

The analysis of tocopherols from SBP extracts was performed using the same method as for phytosterols, described in Section 2.7.

### 2.9. Antimicrobial Activity

#### 2.9.1. The Cultures and the Growth Conditions

The microorganisms used for the experimental tests included Gram-positive bacteria: *Bacillus subtilis* ATCC 6633, *Staphylococcus aureus* ATCC 25923 and *Listeria monocytogenes* Scott A, as well as Gram-negative bacteria: *Pseudomonas fluorescens* ATCC 13525, *Aeromonas hydrophila* ATCC 7966 and *Escherichia coli* ATCC 25922. Selected fungal strains, *Aspergillus niger* and *Penicillium expansum*, from the Microorganism Collection (coded MIUG), Dunărea de Jos University of Galati, were tested for antifungal activity of the enriched SBP extracts. The bacterial strains were grown at 37 °C for 18 h on different media: Brain Heart Infusion Broth (Oxoid, Hampshire, UK) for *Listeria monocytogenes* and Muller-Hinton Broth (Scharlau, Barcelona, Spain) for *E. coli*, *S. aureus*, *Bacillus subtilis* and *Pseudomonas fluorescens*. The fungal strains were grown on Rose-Bengal Chloramphenicol medium (Oxoid, Hampshire, UK).

#### 2.9.2. Antibacterial Activity

The antibacterial effect was assessed in vitro using the agar well diffusion test [17,18]. Fifteen milliliters of a specific culture medium with agar, fluidized and tempered at 45 °C, was poured into sterile Petri dishes. Ten microliters from all working cultures (~10^9^ CFU/mL) was inoculated in 7 mL of molten agar medium (42 °C) and then spread onto the surface of the solidified medium from the Petri dishes, in order to obtain a final concentration of ~10^7^ CFU/plate. After homogenization and medium solidification, wells with a diameter of 9 mm were formed. The extracts were dissolved in the solvent mixture of hexane/acetone (1:3) at a ratio of 1:1, and then 100 µL was placed into the wells. The positive controls were erythromycin (10 mg/mL) for *Staphyllococcus aureus* and *Listeria monocytogenes* and ampicillin (10 mg/mL) for *Escherichia coli*. The solvent mixture of hexane/acetone (1:3) was considered as a negative control. Testing plates were then incubated at 37 °C for 24 h, and the inhibition zones’ diameter was measured (D_iz_, mm).

#### 2.9.3. Antifungal Activity

The antifungal activity was assessed by testing fractions of SBP oleoresins, in the subsequent stages, on two test strains (*Aspergillus niger* and *Penicillium expansum*). The method used was described by Cortes-Zavaleta et al. [19] and Radu et al. [20], with some changes. An amount of 100 µL of extract was homogenized with 9 mL of sterile Rose-Bengal Chloramphenicol Agar culture medium and distributed into Petri dishes. The resulting medium was centrally inoculated with a 10 µL spore suspension with a 1 × 10^4^ spores/mL concentration and incubated at 25 °C for 3–5 days. As a control, plates containing Rose-Bengal medium were prepared with the solvent mixture of *n*-hexane/acetone (1:3) in the same proportions as above. After 3–5 days of incubation, the mycelial growth diameters of the resulting colonies for both treated (*D_t_*) and control (*D_c_*) plates were measured. The growth inhibition rate (*IR*) was calculated using the following Equation (2):

(2) $$IR\left(\%\right)=\frac{\left(D_{c}-D_{t}\right)}{D_{c}}$$

### 2.10. Statistical Analysis

The results are expressed in terms of the average followed by standard deviation. The statistical evaluation was performed using Minitab 19 software. First, the data were checked for normality (Ryan-Joiner test) and homoscedasticity (Bartlett test) conditions. The differences between samples were analyzed using the ANOVA method, and posthoc analysis was performed based on the Tukey or Games-Howell method.

## 3. Results

### 3.1. Influence of SFE-CO_2_ Extraction Parameters on Extraction Yields and Global Phytochemical Profile of Sea Buckthorn Pomace Extracts

Sea buckthorn is a multi-purpose plant and is processed in a wide variety of food products (juice, drink, smoothie, jam, sauce, oil) and alcohols (wine, liqueur, beer additive) from berries, whereas it can also be used as herbal leaf teas to provide high access to flavonoids with detoxifying properties, fuel as firewood, food supplements, etc. [21]. Processing sea buckthorn into a wide range of products leads to a significant amount of by-products (pulp, press cake, pomace) that are considered a valuable source for bioactive extraction. The resulting by-products can be used as natural food coloring or for the production of fodder, tea-type infusions, powders, nutraceuticals and antioxidant additives, as well as an unconventional source of oleoresins with a potential use in the food, cosmetic and pharmaceutical industries [22,23]. In our study, SBP was used as a raw material for SFE-CO_2_ extraction of valuable oleoresins. The application of CO_2_ as an extraction solvent is based on several advantages such as its nontoxicity, non-flammability and low cost, allowing for obtaining high-purity extracts. However, the ability of CO_2_ to dissolve various substances highly depends on the process parameters [11]. In our study, the impact of two independent variables, namely, temperature (*T*) and pressure (*P*), on obtaining enriched oleoresin extracts from SBP was studied. Two non-denaturing temperatures were selected, namely, 35 °C and 37.5 °C, whereas the pressure values were 45 MPa and 36.5 MPa, respectively. Under the applied extraction conditions, the resulting extracts presented similar yields of 67.6 g/kg DW for E1 and 63.6 g/kg DW for E2. Therefore, it can be appreciated that both selected parameters yielded similar extraction yields. Kitrytė et al. [24] used a three-step fractionation process to recover valuable compounds from sea buckthorn pomace and seeds, using successive SFE-CO_2_, pressurized ethanol and enzyme-assisted extraction, finding that SFE-CO_2_ yielded 146 and 135 g/kg of the lipophilic fraction from the pomace and seeds, respectively. The use of the SFE-CO_2_ method to extract bioactives from SBP yielded a variable content of the recovered oil in the range of 5.3–19.5%, depending on the berry cultivar and some other factors [8]. Dienaitė et al. [25] reported a higher extraction yield of 16.99% in oil, at a low rate of CO_2_ of 2 L/min, pressure of 50 MPa and extraction temperature of 50 °C.

In our study, SFE-CO_2_ extraction of oleoresins from SBP allowed obtaining four fractions according to E1 and E2 in the two separators. The global phytochemical profiles of the four fractions are presented in Table 1. The highest carotenoid content was found in E1S40, with 396.12 ± 1.02 mg/g DW, compared with 202.73 ± 0.32 mg/g DW in E2S40. The same trend was observed regarding the β-carotene and lycopene contents, the selected bioactives having a significantly higher concentration in the extracts obtained from E1 (*p* < 0.001). In contrast, no significant difference was observed in the polyphenolic content (33.89 ± 0.58 mg GAE/g DW and 33.64 ± 0.58 mg GAE/g DW, respectively) (Table 1). The carotenoid content in E1S40 displayed a significantly higher antioxidant activity of 32.10 ± 0.17 mM TEAC/g DW compared with 21.50 ± 0.11 mM TEAC/g DW in E2S40. When considering the second separator extracts (ES45), it can be observed from Table 1 that the carotenoid content in terms of total carotenoid, β-carotene and lycopene contents was approximately two times higher in E2 when compared with E1.

However, no significant differences (*p* > 0.05) were found in polyphenols. Significant differences (*p* < 0.001) were found in the antioxidant activity, with values of 14.25 ± 0.09 mM TEAC/g DW for E1S45 and 3.22 ± 0.009 mM TEAC/g DW for E2S45. The highest pressure (45 MPa) and lowest temperature (35 °C) returned a higher carotenoid content and antioxidant activity in the first fraction of extracts, whereas increasing the temperature to 37.5 °C and lowering the pressure to 36.5 MPa resulted in enriched ES45 fractions, with a higher content of carotenoids, but a lower antioxidant activity. These results are associated with the density of CO_2_ which increases at a higher pressure and lower temperature, leading to higher diffusivity and solvating power [26]. Overall, the extraction parameters allowed identifying more than thirty carotenoids with a major concentration in E2 and a better extraction of zeaxanthin (E2) and β-carotene (E1). A maximum recovery of 77.2% of tocopherol and 75.5% of carotene and an EC50 of 49.9 mg/mL (from the DPPH assay) were obtained by Kagliwal et al. [27], after SC-CO_2_ extraction at 35 °C, 400 bar, for 60 min.

Dąbrowski et al. [28] studied the carotenoid content in five cultivars and reported contents varying from 0.67 (Luczystaja cv. L) to 1.41 mg/g (Golden Rain 296 cv.), with an average value of 1.16 mg/g. Tkacz et al. [21] studied the total phenolic compounds in seven cultivars and reported values ranging from 0.51 in the flesh to 121.97 mg GAE/g DW in the branches.

### 3.2. The Individual Carotenoid Profiles of the Enriched Oleoresin Extracts

**Figure 1:**
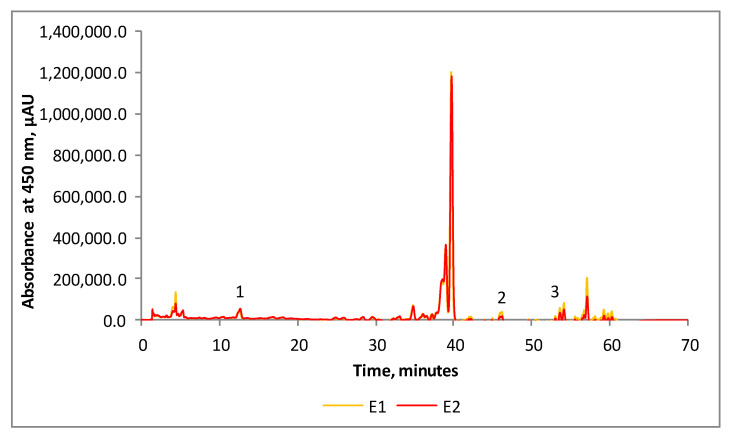
Chromatographic profile of carotenoids from SBP oleoresins (peak 1—zeaxanthin; peak 2—lycopene; peak 3—β-carotene).

The analyzed samples were characterized in detail by chromatographic techniques. The chromatographic profile of carotenoids from the enriched oleoresin extracts is represented in Figure 1. We were able to separate and identify carotenoids from both samples, but due to the small quantity of each sample, we decided to combine both fractions, S40 and S45, of each extract. The analyzed samples contained thirty-eight (E1) and thirty (E2) carotenoid compounds. Only three of them were quantified according to the available standards in our laboratory: peak 1, zeaxanthin, 19.08 μg/mL (E1) to 20.64 μg/mL (E2); peak 2, lycopene, 2.08 μg/mL (E1) to 2.85 μg/mL (E2); and peak 3, β-carotene, 6.57 μg/mL (E2) to 10.47 μg/mL (E1).

Madawala et al. [29] suggested that in sea buckthorn extract, four carotenoids predominate, namely, β-carotene, lycopene, zeaxanthin and lutein, with significant differences between cultivars and geographical regions. However, Gheonea et al. [16] found that nine carotenoids, namely, all-trans-β-carotene, β-cryptoxanthin, lutein, zeaxanthin, zeinoxanthin, α-cryptoxanthin, α-carotene, 9-cis-β-carotene, γ-carotene and lycopene, prevailed, ranging from 11.7% to 17.0% and from 4.9% to 14.5%.

### 3.3. The Individual Fatty Acid Profiles of the Enriched Oleoresin Extracts

Eleven fatty acids were identified in all four fractions of enriched oleoresin extracts including myristic acid (C14:0), pentadecanoic acid (C15:0), palmitic acid (C16:0), palmitoleic acid (C16:1), stearic acid (C18:0), oleic acid (C18:1 (n9)), linoleic acid (C18:2), γ-linolenic acid (C18:3), eicosaenoic acid (C20:1), eicosadienoic acid (C20:2) and eicosapentanoic acid (C20:5). The results of the quantified individual fatty acids, saturated fatty acids (SFAs), monounsaturated fatty acids (MUFAs) and polyunsaturated fatty acids (PUFAs) are presented in Table 2. Therefore, in E1S40 and E1S45, linoleic acid (30.28% and 31.11%, respectively), γ-linolenic acid (21.40% and 22.63%, respectively), oleic acid (17.29% and 14.27%, respectively), palmitic acid (15.63% and 16.77%, respectively) and palmitoleic acid (10.71% and 10.99%, respectively) were the major fatty acids in the SBP oleoresins, representing more than 95% of the total fatty acid content. E2 had the same predominant fatty acids as E1, with slight differences in their concentrations. Thus, in E2S40 and E2S45, the proportion of linoleic acid was 27.03% and 29.88%, respectively; for γ-linolenic acid, it was 19.70% and 21.62%, respectively; for palmitic acid, it was 19.12% and 17.99%, respectively; for oleic acid, it was 18.59% and 14.84%, respectively; and for palmitoleic acid, it was 10.77% and 11.65%, respectively. A comparison between the two sets of extractions revealed major differences both in oil yields and fatty acid profiling, with an oil yield of 606.02 mg/g for E1S40, 536.03 mg/g for E1S45, 490.54 mg/g for E2S40 and 525.76 mg/g for E2S45 (Table 2).

In all fractions, high amounts of palmitoleic acid (C16:1) were found, in concentrations ranging from 52.9 to 65 mg/g. Palmitoleic acid belongs to the ω-7 group, is a component of skin lipids and may act as a regulator in the process of epidermis regeneration. In addition, it was reported that palmitate-induced macrophage activation and skeletal muscle insulin resistance could be diminished by the presence of palmitoleic acid [30]. Additionally, Tricò et al. [31] showed the beneficial effect of palmitoleate as an adipocyte-derived lipid hormone (lipokine) released to prevent the harmful effects of adiposity and excess non-esterified fatty acids on systemic glucose metabolism. Additionally, from Table 2, it should be noted that more than 50% and 46–51% of the extracts obtained in E1 and E2, respectively, were represented by two PUFAs, namely, C18:2 and C18:3, whereas stearic acid (C18:0) concentrations varied from 2.62% and 3.26%.

In all enriched oleoresin extracts, the SFA, MUFA and PUFA ratios were 19.60%, 28.38% and 52.00% in E1S40, and 20.33%, 25.56% and 54.10% in E1S45, whereas in E2S40 and E2S45, values of 23.11%, 29.75% and 47.13% and 21.37%, 26.76% and 51.85%, respectively, were found. Other minor fatty acids varied from 0.15% (C20:2) to 0.38% (C20:1), representing less than 0.7% of the total fatty acid content. The saturated-to-unsaturated fatty acid ratio varied from 1:3.3 in E2S45 to 1:4.1 in E1S40.

Yang et al. [32] studied the fatty acid contents of sea buckhorn pulp and seed oils extracted by supercritical CO_2_ and found a content of 38.6% of palmitoleic acid, 29.1% of palmitic acid and 16.6% of oleic acid in the pulp oil, whereas linoleic acid (37.4%), α-linolenic acid (29.0%) and oleic acid (20.3%) were predominant in the seed oil. Gutiérrez, Ratti and Belkacemi [33] suggested that PUFAs amounted to between 70% and 75% of the total fatty acids in the seed oils, while MUFAs and SFAs amounted to about 18% and 11%, respectively.

### 3.4. The Individual Tocopherol and Phytosterol Profiles of the Enriched Oleoresin Extracts

In addition to the remarkable high shares of PUFAs and, in particular, ω-7 palmitoleic acid and carotenoids, sea buckthorn SFE-CO_2_ extracts were abundant in sterols and tocopherols. All enriched SFE-CO_2_ extracts contained three monomers, α-, β- and δ-tocopherol, with α-tocopherol being the major compound, representing about 82–84% of all fractions. The results obtained in this study are significantly higher than those reported by Dienaitė et al. [25], who found a concentration of 117.58 mg/100 g DWE of α-tocopherol in the SFE-SBP oil, representing up to 68% of all tocopherols. The abundance of α-tocopherol in all the SFE-CO_2_ extracts obtained from SBP has a particular importance, since it was recently clarified that vitamin E activity may be assigned only to α-tocopherol, due to its ability to prevent the human deficiency disease “ataxia with vitamin E deficiency” [34]. Ranjith et al. [35] identified seven monomers (including α-, β-, γ- and δ-tocopherol and α-, γ- and δ-tocotrienol), whereas the sum of these compounds in the sea buckthorn pulp oils ranged from 66.6 to 178.8 mg/100 g.

**Figure 2:**
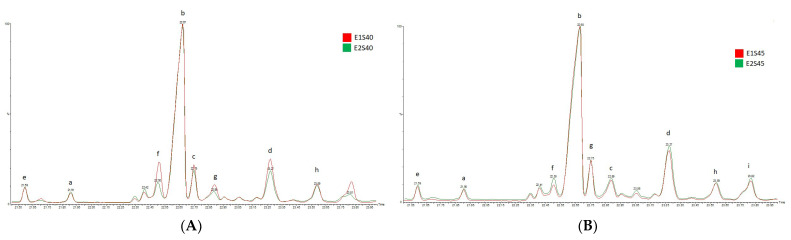
Phytosterol composition of SFE-CO_2_ SBP oil fractions (a—campesterol; b—β-sitosterol; c—β-amyrin; d—α-amyrin; e–i—not identified). (**A**) fractions derivated from SFE-CO_2_ extraction at 35 °C/45 MPa (E1) and 37.5 °C/36.5 MPa (E2), respectively, in separator S45, (**B**) fractions derivated from SFE-CO_2_ extraction at 35 °C/45 MPa (E1) and 37.5 °C/36.5 MPa (E2), respectively, in separator S40.

In the SFE-CO_2_ SBP extracts, four major compounds were identified: campesterol, β-sitosterol, β-amyrin and α-amyrin. β-sitosterol was the dominant component in all enriched fractions, with the highest concentration in E2S45 (Figure 2A). β-sitosterol was found in the highest concentration in the fraction derived from S45, representing 95.20% of the total phytosterols in E1S45 and 87.90% in E2S45. The SBP fractions separated in S40 showed a significantly lower concentration, representing 83.41% in E1S40 and 78.03% in E2S40 (Figure 2B).

Zheng et al. [36] found that total phytosterol concentrations in sea buckthorn oils ranged from 778 ± 70 to 1847 ± 29 mg/100 g. In contrast, Li et al. [37] identified ten phytosterols in sea buckthorn seed oils, namely, campesterol, lanosterol, sitosterol, β-amyrin, sitostanol, α-amyrin, lupeol, cycloartenol, erythrodiol and uvaol. These authors tested different extraction methods and reported that SFE allowed obtaining the highest amount of total phytosterols, followed by hexane and cold press extractions, with 1640 mg/100 g oil, 1326 mg/100 g oil and 879 mg/100 g oil, with β-sitosterol and Δ^5^-avenasterol having the highest concentrations.

Remarkably, in our study, the β-sitosterol concentrations (ranging from 78 to 83% in S40 fractions, and from 88% to 95% in S45 fractions) were significantly higher than those from other reports (57–76%) [38]. The abundance of phytosterols in the obtained extracts has a particular importance, as the health-promoting properties of these bioactives have been reported in many articles, which are based on the structural similarity of phytosterols to cholesterol. Therefore, due to these similarities, phytosterols reduce intestinal cholesterol absorption, thus alleviating blood LDL cholesterol and cardiovascular problems. Additionally, it has been reported that phytosterol-rich diets may reduce cancer risk by a significant 20% [39]. Given the obtained results, all four SFE-CO_2_ extracts from SBP showed a satisfactory content of sterols, representing a promising source of bioactives in developing healthy foods.

### 3.5. Antimicrobial Activity

**Figure 3:**
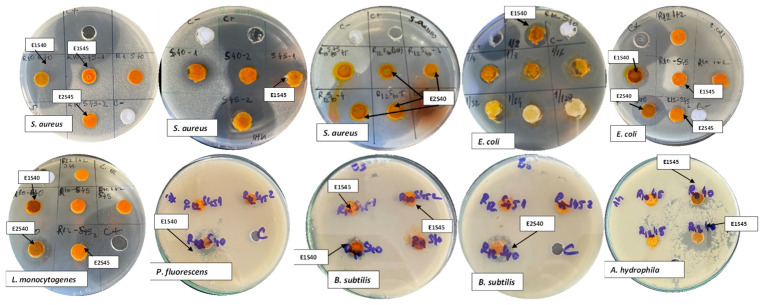
Zones of inhibition of SFE-CO_2_ extracts against various bacterial strains.

Various studies have reported the antibacterial activity of aqueous and hydroalcoholic extracts from different parts of the sea buckthorn plant against pathogenic and/or facultative pathogenic bacteria. However, limited information is available on the antimicrobial activity of SFE-CO_2_ SBP extracts. The SBP extracts resulting from SFE-CO_2_ extraction inhibited the growth of the foodborne bacteria tested in this study. After 24 h of cultivation, the results were obtained by analyzing the diameters of the growth inhibition zones of the tested strains (Table 3). The results indicate a good antibacterial activity of the extracts, especially for the fractions obtained in S40. *Staphylococcus aureus* (E1S40 27.35 ± 0.35 mm, and E2S40 21.40 ± 0.28 mm) and *A. hydrophila* (E2S45 20.33 ± 1.15 mm) showed a relatively high inhibition area, while *P. fluorescens* showed higher resistance to the tested extracts. The resistance or susceptibility of the bacterial strains towards SFE-CO_2_ extracts was determined by measuring the zones of inhibition, as presented in Figure 3.

Arora et al. [40] tested the antibacterial properties of methanolic and aqueous extracts from various parts of sea buckthorn (pulp, seeds, leaves and pomace) on 17 pathogens that induce a food safety risk, including *B. cereus* ATCC 11778, *L. monocytogenes* ATCC 15313, ATCC 19111, *S. aureus* ATCC 12598, ATCC 12600, *A. hydrophila* ATCC 7966, *E. coli* ATCC 8739, ATCC 25922 and *P. fluorescens* ATCC 13525. Similar to our results, *S. aureus* was the most sensitive, with an inhibition zone equal to 13.30 ± 1.50 mm for the extracts obtained from pomace. Positive results were also obtained by Radenkovs et al. [23], who reported significant antimicrobial activity of *H. rhamnoides* L. against aerobic or facultative anaerobic Gram-positive bacteria and Gram-negative pathogenic bacteria, which included *Bacillus* spp., *Salmonella* spp., *E. coli*, *Yersinia pestis*, *Klebsiella* spp. and *Shigella* spp. Significant antibacterial activity was recorded for sea buckthorn juice against different strains of *Streptococcus* spp., *Staphylococcus* spp. and *Pseudomonas* spp. at a juice concentration of over 20% [41].

The antifungal effect of the tested extracts is shown in Table 4. It can be seen that a growth inhibition rate (IR) of 100% was found for E2S45 against *Aspergillus niger* after three days of growth, decreasing to 73.88% after five days. A higher IR for *Penicillium expansum* was obtained after five days for E1S45 (IR = 21.93%). Except for E2S45, the most resistant fungal strain was *Aspergillus niger* for all extracts, showing low antifungal activity.

Similar results were obtained by Czerwińska and Szparaga [42], who reported antimicrobial activity of some extracts of *Lavandula vera* L. against *Aspergillus glaucus* and *Aspergillus niger*. From the selected strains, *Aspergillus niger* had the highest resistance. 

The variable antimicrobial activity of the extracts from sea buckthorn fruits and leaves may be related to the bioactive compounds present in the plant, especially carotenes, polyphenols, flavonoids [43,44] and polyunsaturated fatty acids, mainly linoleic acid and oleic acid, for Gram-positive bacteria [45,46]. The mechanism of antimicrobial activity refers to the precipitation of membrane proteins, leading to cell lysis [20]. However, the effects of fatty acids on the molecular structure of the cytoplasmic membrane could be explained by their introduction into the phospholipid bilayer of the membrane, leading to destabilization of the membrane [20].

## 4. Conclusions

Different parameters in terms of temperature and pressure were tested for supercritical fluid extraction of sea buckthorn pomace using CO_2_, allowing for obtaining four lipophilic fractions. These fractions were analyzed, and the results highlight significant differences both in the phytochemical content and selected functionality. The pressure had a significant influence on the extract profiles. In summary, the extracts obtained at an extraction temperature of 35 °C and pressure of 46.5 MPa showed a higher content of bioactives, such as carotenoids, β-carotene, β-tocopherol, β-sitosterol and polyunsaturated fatty acids. All fractions presented a unique composition and concentration of bioactives, with a particular emphasis on the palmitoleic acid content. Significant antioxidant and antimicrobial activities were found for the fractions obtained in the first separator at the selected extraction parameters.

The results of this study are valuable, and they allow controlling the phytochemical profile, both from qualitative and quantitative perspectives, and thus obtaining enriched extracts with selected functional and biological properties. These extracts recovered from sea buckthorn berry pomace by green supercritical extraction with CO_2_ techniques are promising substances for further development of functional ingredients for various applications, particularly in functional foods and nutraceuticals.

## Figures and Tables

**Table 1:** Phytochemical composition of SFE-CO_2_ SBP oil fractions.

Phytochemical Content	E1		E2	
	S40	S45	S40	S45
Total carotenoid content, mg/g DW	396.12 ± 1.02 ^a^	56.34 ± 0.12 ^b^	202.73 ± 0.32 ^b^	96.49 ± 0.15 ^a^
β-carotene content, mg/g DW	321.35 ± 0.35 ^a^	50.68 ± 0.18 ^b^	185.95 ± 1.94 ^b^	94.64 ± 0.11 ^a^
Lycopene, mg/g DW	66.82 ± 1.10 ^a^	16.81 ± 0.90 ^b^	50.32 ± 2.81 ^b^	31.58 ± 0.08 ^a^
Polyphenols, mg GAE/g DW	33.89 ± 0.58 ^a^	8.70 ± 0.44 ^a^	33.64 ± 0.58 ^a^	8.72 ± 0.44 ^a^
Antioxidant activity, mM TEAC/g DW	32.10 ± 0.17 ^a^	14.25 ± 0.09 ^a^	21.50 ± 0.11 ^b^	3.22 ± 0.009 ^b^

Average values in the same row and same fraction (S40 or S45) that do not share the same lowercase letter (a, b) are statistically significant at *p* < 0.001, based on the Games-Howell method or Tukey method and 95% confidence.

**Table 2:** Fatty acid and carotenoid profiles of the SFE-CO_2_ SBP oil fractions.

Compound	E1		E2	
	S40	S45	S40	S45
Fatty Acids (mg/g)				
Myristic acid (C14:0)	3.00 ± 0.98	3.02 ± 0.75	2.36 ± 0.54	2.70 ± 0.41
Pentadecanoic acid (C15:0)	1.43 ± 0.12	1.35 ± 0.11	1.19 ± 0.09	1.29 ± 0.04
Palmitic acid (C16:0)	94.80 ± 1.23	89.90 ± 1.78	93.80 ± 3.09	94.60 ± 2.01
Palmitoleic acid (C16:1)	65.00 ± 2.35	58.90 ± 3.22	52.90 ± 2.11	61.30 ± 1.12
Stearic acid (C18:0)	19.60 ± 1.45	14.73 ± 1.89	16.02 ± 1.06	13.80 ± 1.09
Oleic acid (C18:1)	104.80 ± 12.35	76.40 ± 3.78	91.20 ± 2.01	78.00 ± 3.45
Linoleic acid (C18:2)	183.50 ± 10.97	166.80 ± 12.78	132.60 ± 9.89	157.10 ± 6.54
γ-Linolenic acid (C18:3)	129.70 ± 5.67	121.30 ± 4.32	96.70 ± 3.21	113.70 ± 7.68
Gondoic acid (C20:1)	2.24 ± 0.64	1.73 ± 0.99	1.90 ± 0.89	1.44 ± 0.78
cis-11,14-Eicosadienoic acid (C20:2)	0.96 ± 0.03	0.91 ± 0.09	0.91 ± 0.09	0.88 ± 0.05
cis-5,8,11,14,17-Eicosapentanoic acid (C20:5)	0.99 ± 0.05	0.99 ± 0.07	0.96 ± 0.08	0.95 ± 0.03
Total fatty acids, mg/g	606.02	536.03	490.54	526.76

**Table 3:** Antibacterial effect of the tested extracts.

	Diameter of Inhibition Zone (mm)					
Test Compound/Bacterial Strain	*E. coli*	*S. aureus*	*L. monocytogenes*	*B. subtilis*	*P. fluorescens*	*A. hydrophila*
Control	0	0	0	0	0	0
E1S40	16.75 ± 0.35 ^a^	27.35 ± 0.35 ^a^	0 ^c^	14.33 ± 0.58 ^b^	0 ^b^	10.67 ± 0.58 ^b^
E1S45	0 ^b^	10.15 ± 0.21 ^c^	18.10 ± 0.14 ^a^	9.67 ± 0.58 ^c^	0 ^b^	0 ^c^
E2S40	16.25 ± 0.35 ^a^	21.40 ± 0.28 ^b^	13.50 ± 0.7 ^b^	17.67 ± 0.58 ^a^	18.33 ± 2.08 ^a^	20.33 ± 1.15 ^a^
E2S45	0 ^b^	0 ^d^	0 ^c^	0 ^d^	0 ^b^	0 ^c^

Average values in the same column that do not share the same lowercase letter (a, b, c) are statistically significant at *p* < 0.001, based on the Games-Howell method or Tukey method and 95% confidence. ANOVA and posthoc analyses did not include control sample.

**Table 4:** Antifungal effect of tested extracts.

Extracts	Mycelial Growth Diameter (mm)		IR (%)	
	3 Days	5 Days	3 Days	5 Days
	*Aspergillus niger*			
Control	51.88 ± 0.18 ^a^	61.75 ± 0.35 ^a^	-	-
E1S40	43.46 ± 0.29 ^b^	48.38 ± 0.18 ^d^	16.22 ± 0.85 ^b^	21.66 ± 0.73 ^b^
E1S45	50.63 ± 0.17 ^a^	60.00 ± 0.00 ^b^	2.41 ± 0.01 ^c^	2.83 ± 0.56 ^d^
E2S40	43.63 ± 1.24 ^b^	55.50 ± 0.71 ^c^	16.11 ± 2.38 ^b^	10.08 ± 0.57 ^c^
E2S45	0.00 ^c^	16.13 ± 0.53 ^e^	100.00 ^a^	73.88 ± 1.01 ^a^
	***Penicillium expansum***			
Control	20.88 ± 0.18 ^a^	26.75 ± 0.35 ^a^	-	-
E1S40	20.17 ± 0.24 ^ab^	23.83 ± 0.24 ^b^	3.39 ± 0.31 ^b^	10.90 ± 0.30 ^ba^
E1S45	18.88 ± 0.18 ^ab^	21.00 ± 0.00 ^c^	9.58 ± 0.08 ^ab^	21.49 ± 1.04 ^a^
E2S40	18.25 ± 1.06 ^b^	24.13 ± 0.18 ^b^	12.59 ± 4.34 ^a^	10.66 ± 0.65 ^b^
E2S45	19.75 ± 0.35 ^ab^	20.88 ± 0.88 ^c^	5.39 ± 0.89 ^ab^	21.93 ± 4.34 ^a^

Average values in the same column that do not share the same lowercase letter (a, b, c) are statistically significant at *p* < 0.001, based on the Games-Howell method or Tukey method and 95% confidence.

## Data Availability

Data are contained within the article.
